# Dengue-induced autophagy, virus replication and protection from cell death require ER stress (PERK) pathway activation

**DOI:** 10.1038/cddis.2015.409

**Published:** 2016-03-03

**Authors:** E Datan, S G Roy, G Germain, N Zali, J E McLean, G Golshan, S Harbajan, R A Lockshin, Z Zakeri

**Affiliations:** 1 Department of Biology, Queens College and Graduate Center of the City University of New York, Flushing, NY, USA

## Abstract

A virus that reproduces in a host without killing cells can easily establish a successful infection. Previously, we showed that dengue-2, a virus that threatens 40% of the world, induces autophagy, enabling dengue to reproduce in cells without triggering cell death. Autophagy further protects the virus-laden cells from further insults. In this study, we evaluate how it does so; we show that dengue upregulates host pathways that increase autophagy, namely endoplasmic reticulum (ER) stress and ataxia telangiectasia mutated (ATM) signaling followed by production of reactive oxygen species (ROS). Inhibition of ER stress or ATM signaling abrogates the dengue-conferred protection against other cell stressors. Direct inhibition of ER stress response in infected cells decreases autophagosome turnover, reduces ROS production and limits reproduction of dengue virus. Blocking ATM activation, which is an early response to infection, decreases transcription of ER stress response proteins, but ATM has limited impact on production of ROS and virus titers. Production of ROS determines only late-onset autophagy in infected cells and is not necessary for dengue-induced protection from stressors. Collectively, these results demonstrate that among the multiple autophagy-inducing pathways during infection, ER stress signaling is more important to viral replication and protection of cells than either ATM or ROS-mediated signaling. To limit virus production and survival of dengue-infected cells, one must address the earliest phase of autophagy, induced by ER stress.

*Flaviviridae* includes some of the most deadly human viruses including yellow fever, west Nile, hepatitis C and dengue,^1^ and one approach of controlling them is to restrict their reproduction in humans. Dengue is endemic in ∼100 countries with 40% of the global population susceptible to infection. Infection has doubled over the past two decades, currently totaling 50–100 million per year.^2^

These viruses regulate the metabolism and survival of infected cells, assuring their own reproduction and propagation. Dengue infection also triggers autophagy, a general homeostatic response that helps the infected cell survive and produce virus.^3, 4, 5^ Here we report that dengue virus induces autophagy through activation of endoplasmic reticulum (ER) stress and ataxia telangiectasia mutated (ATM) signaling and the production of reactive oxygen species (ROS), enhancing its ability to reproduce.

Our laboratory and others have demonstrated that dengue virus induces autophagy and protects cells against other stressors.^4, 5^ We have attributed the protection of infected cells to the induction of autophagy, and proved the involvement of the viral NS4A (nonstructural protein 4A) protein in these events.^4^ Inhibition of dengue-induced autophagy by pharmacological inhibitors or deficiency of autophagy-related genes (ATG) reduces dengue replication and leads to temperature-sensitive, mutant virions.^5, 6, 7^ An understanding of virus-regulated autophagy will enable us to limit the impact of infection.

We briefly summarize below the primary pathways that regulate autophagy. Autophagy is a highly conserved catabolic process involving the transport of proteins, lipids, organelles to double-membraned vesicles (autophagosomes) and thence to the lysosome for subsequent degradation (see review, see Yorimitsu and Klionsky^8^). The formation and expansion of the autophagosome is governed by several complexes of molecules, including the ULK1 (*U*nc-*l*ike *k*inase) complex, the beclin-1–VPS34 (vacuolar protein-sorting protein 34; class III phosphatidylinositol 3-kinase)–AMBRA1 (autophagy/beclin-1 regulator) complex, the ATG9–WIPI (WD repeat domain, phosphoinositide interacting/ATG 18 homolog) transmembrane complex and the ubiquitin-like ATG12–LC3 (microtubule-associated protein 1 light chain 3) complex.^9^ The prime mediators of initiation or induction of autophagy comprise sensors of cell energy (AMPK (adenosine monophosphate-activated protein kinase) and nutrition (mTOR (mammalian target of rapamycin)). Under normal conditions, mTOR blocks autophagy through phosphorylation of ULK1 (at Ser 757) and ATG13, whereas AMPK (lying upstream of mTOR) can remove mTOR suppression during stress such as starvation and induce autophagy.^10, 11, 12, 13^ An upstream modulator of AMPK – ATM, a nuclear protein involved in DNA damage response (DDR) – is also a positive effector of autophagy.^14^ As a key surveillance protein in the cell cycle, ATM kinase has ancillary functions in chromatin organization, gene expression and DNA/RNA/protein metabolism.^15^ ATM is exported to the cytoplasm in response to high concentration of ROS and reactive nitrogen species (RNS) like nitric oxide (NO), and deactivates mTOR through a series of phosphorylation-dependent activation processes involving liver kinase B1 (LKB1), AMPK and the tumor suppressor tuberous sclerosis 2 (TSC2).^12, 14, 16, 17, 18^

ER stress is linked to ROS-mediated autophagy. Monocyte chemotactic protein-1 (MCP-1), known to mediate cardiac injury, induces ROS, ER stress and autophagy in cardiac myoblasts (H9c2 cells).^19^ In most cases, as in mouse disease models, induction of autophagy by ER stress serves as a protective mechanism against apoptotic cell death.^20^ One of the most important branches of ER stress/unfolded protein response (UPR) signaling – eukaryotic translation initiation factor-2*α* (eIF2*α*/protein kinase R-like endoplasmic reticulum kinase (PERK)) – is activated in response to accumulation of proteins with polyglutamine repeats and functions in LC3 lipidation and autophagosome formation.^21^ PERK-induced autophagy also protects mouse and human lymphomas during pathogenic conditions (Myc-induced tumorigenesis).^22^ Downstream ER stress components like C/EBP homologous protein (CHOP), a PERK-regulated protein, and inositol-requiring protein 1 (IRE1) also increase autophagy in colon cancer cell lines like HT29 (human colon adenocarcinoma cell line), SW480 (human colorectal adenocarcinoma cell line) and Caco-2,^23^ demonstrating the involvement of ER stress signaling in the induction of autophagy.

Here we show that although many autophagy-inducing pathways are activated in dengue-infected cells, inhibition of ER stress signaling limits the ability of dengue-2 virus to induce autophagy and protect infected cells. In contrast, protection of virus-laden cells from inducers of apoptosis by increasing autophagy increases the potential of dengue to replicate within cells and establish successful infections.

## Results

### ER stress signaling, activated during infection, is required for virus-induced autophagy, protection of cells and production of virus

**Figure 1:**
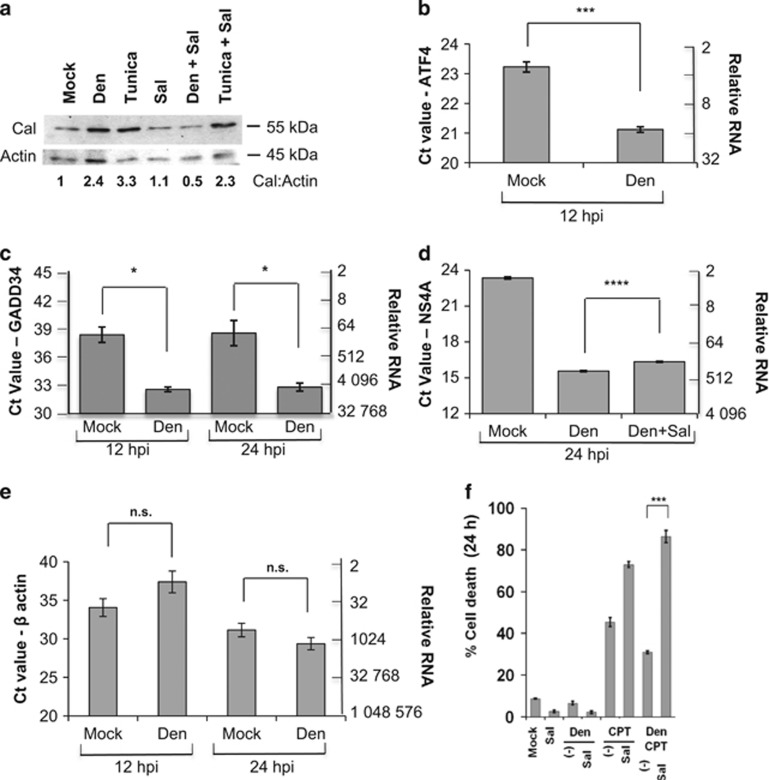
Dengue induces ER stress, especially activating the PERK pathway, accelerating its replication and protecting MDCK cells from toxicity of CPT. (**a**) A general marker of ER stress, calreticulin, increases during infection (dengue (Den)) or treatment with ER stress inducer tunicamycin (Tunica), whereas the inhibitor of ER stress response, salubrinal (Sal), decreases calreticulin even in infected cells (Den+Sal) and modestly suppresses the response to tunicamycin. Numbers below blot represent protein ratios to loading control. The cells were treated and/or infected for 24 h before the proteins were isolated for immunoblotting. (**b**) Dengue infection in MDCK significantly increases ATF4 gene expression at 12 h. (**c**) Transcription of GADD34, a downstream target of CHOP, increases at 12 HPI and remains high, relative to actin (control, illustrated in Figure 1e). (**d**) At 24 h of infection, salubrinal depresses the transcription of dengue NS4A gene by 42% (only 129-fold greater than mock for Den+Sal compared with 222-fold greater than mock for Den). Compared with mock, for transcription of NS4A, *P*<0.0001. Compared with infected cells without Sal, production of NS4A was 42% less, *P*<0.0004. (**e**) Verification of controls: transcription of *β*-actin in mock- and Den-infected MDCK cells after 12 and 24 h of infection. Infection has minimal and insignificant (n.s.) impact on the transcription of actin (**f**). In the presence of Sal, an inhibitor of the PERK pathway, there was higher cell death in CPT-treated and Den-infected MDCK cells after 24 h. In this and subsequent figures, *P<0.05; **P<0.01, ***P<0.005 and ****P<0.001 for the bracketed comparisons

Flavivirus including dengue are ER tropic and cause the ER to initiate stress response signaling.^24^ To ascertain the activation of ER stress, we examined expression of the chaperone protein calreticulin, a marker of global ER stress.^24, 25^ Calreticulin has been used as a positive indicator of ER stress in various cell types and model organisms.^26, 27, 28, 29, 30, 31, 32^ 10^6^ MDCK (Madin Darby canine kidney cell line) cells were infected with dengue virus (multiplicity of infection (MOI)=5) for 24 h and were then lysed for protein extraction and western blotting. Dengue infection increases the amount of calreticulin in infected cells within 24 h compared with mock-infected cells (Figure 1a, Cal). This increase in calreticulin is also observed in MDCK cells treated (24 h) with tunicamycin, a pharmacological inducer of ER stress.^33^

Salubrinal, a specific inhibitor of the PERK pathway, inhibits dengue-induced increase of calreticulin at 24 h post infection (HPI) but only barely reduces the high level of calreticulin induced by tunicamycin (Figure 1a). Numbers below the gels signify the ratios of calreticulin to loading control actin. These results suggest activation of ER stress signaling via the PERK pathway in dengue-infected cells.

To determine whether virus-induced ER stress can be attributed to the PERK pathway – an important component of ER stress-mediated UPR^33^ – we used three markers (Figures 1b, c and 3c) of the PERK pathway: ATF4 (cyclic AMP-dependent transcription factor 4), GADD34 (growth arrest and DNA damage-inducible protein 34) and CHOP.^24, 25^ Dengue infection significantly increases (*P*<0.002) ATF4 transcription (as measured by PCR) after 12 h (Figure 1b), implying PERK activation. GADD34 transcript is also significantly elevated (Figure 1c) at 12 and 24 h after infection. We find no significant change in actin transcription in mock or infected MDCK cells after 12 h (*P*=0.14) and 24 h (*P*=0.2) of infection (Figure 1e). Taken together, these results confirm the activation of ER stress signaling – via the PERK pathway – in dengue-infected cells. For each quantitative PCR (qPCR) data presented in this study, we have added a scale for relative RNA corresponding to threshold cycle (Ct) value.

To evaluate the importance of an active PERK pathway in dengue-induced ER stress (and infection as a whole), we asked how salubrinal affected transcription of dengue genes. Using specific primers (see Materials and Methods) for qPCR, we found that salubrinal decreased transcription of the viral NS4A gene by >40% (Figure 1d), indicating the importance of PERK signaling in virus replication and transcription. Our data may explain earlier reports of reduced dengue infectivity in salubrinal-treated A549 (human alveolar adenocarcinoma cell line) cells after 48 h of infection.^34^

For the past two decades, camptothecin (CPT) has been widely used to induce apoptosis in various cells.^35, 36, 37, 38^ We previously demonstrated a link between induction of autophagy and protection of cells – including MDCK – during dengue infection. We have shown that dengue-induced autophagy leads to cell protection from CPT-induced death.^4^ MDCK cells were treated with 70 *μ*M CPT for 24 h with or without dengue infection, and with or without 24 h of salubrinal treatments. We confirmed that protection – from CPT-induced cell death – in dengue-infected cells was abrogated by salubrinal (Figure 1f). Salubrinal is not toxic by itself or in combination with dengue infection (Figure 1f). Thus, the PERK pathway is an important component linking viral replication, induction of ER stress and resistance to toxins of dengue-infected MDCK cells.

**Figure 2:**
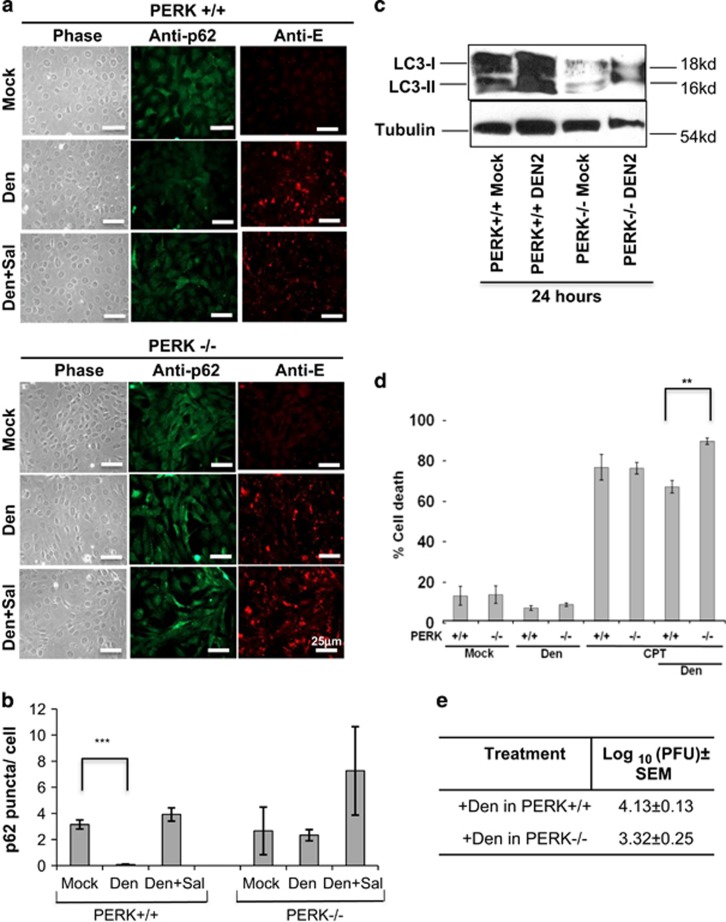
An activated PERK pathway, during dengue infection, is essential for high autophagy turnover in MEF cells. (**a**) Bright-phase micrographs of PERK wild-type (PERK+/+) and PERK KO (PERK−/−) MEFs show adherent cells with minimal cytopathology. Dengue infection in PERK+/+ results in p62 degradation after 48 h, indicating a high autophagosome turnover, whereas treatment with ER stress inhibitor salubrinal (Sal) lowers turnover in infected PERK+/+ cells. We observed a more modest recovery of p62 after infection in PERK−/− cells as compared with the wild-type cells. Anti-dengue E protein (anti-E) signal is present in all infected cells; there is slightly less production in infected PERK−/− and Sal-treated PERK+/+ cells. (**b**) The graph indicating the total number of puncta per cell illustrates a significant decrease (*P*-value: 0.003) in the GFP puncta in infected PERK WT cells and recovered increase in puncta in infected cells exposed to Sal. (**c**) Western blot of whole-cell lysates, obtained after 24 h of mock and dengue (Den) infection, reveals an increase in the LC3 lipidation of PERK+/+ cells. PERK−/− cells, however, do not show a significant LC3 lipidation after 24 h of infection. (**d**) By Trypan blue exclusion assay, we observe a loss after 24 h of infection of dengue-conferred protection against CPT in PERK−/− MEFs compared with wild types. (**e**) Using plaque assay, we compared production of virus in PERK+/+ and PERK−/− cells after 48 h of infection. Dengue plaque formation decreases significantly (85%, 0.8 log_10_ units) in the PERK KO cells. The experiments were repeated at least three times

To further characterize the components of this relationship, we looked at possible links between ER stress (especially PERK pathway,) and turnover of autophagy vacuoles, reflected by levels of lysosomal protein p62 (sequestosome-1/ubiquitin-binding protein), an established marker for autophagy turnover. p62 is degraded as a result of high rate of degradation of autophagosome content in the lysosomes, the final step of autophagy.^39^ We used PERK+/+ (wild type) and PERK−/− (knockout (KO) variants) mouse embryonic fibroblast (MEF) cells to examine the effect of PERK on dengue-induced autophagy (Figure 2a). The p62 degradation was measured in terms of green puncta, as obtained by probing with anti-p62 antibody (AlexaFluor 488). Infected PERK+/+ cells showed a pronounced decrease in p62 compared with mock-infected samples (Figures 2a and b) following 48 h of infection. This decrease in p62 by dengue was not observed in the presence of salubrinal. PERK deficiency reduced autophagy turnover (PERK−/− dengue) compared with infected PERK+/+ cells. We also present a graphical representation of the change in fluorescence signals in mock and infected cells (Figure 2b). The p62-positive puncta were counted from ~200–300 cells for each condition. Our analysis indicated a significant decrease in the puncta count in infected PERK+/+ cells, but no significant difference in infected PERK−/− cells (Figures 2a and b).

Parallel to low autophagy turnover, expression of dengue E protein decreased in salubrinal-treated infected PERK+/+ cells or in infected PERK−/− cells, implying less virus titer (Figure 2a). These results are supported by plaque assay (Figure 2e) demonstrating ~85% decrease of plaque-forming units in infected PERK−/− cells compared with their wild-type counterpart (PERK+/+). In line with this finding, the infected PERK+/+ cells showed increased LC3 lipidation after 24 h of infection, compared with mock-infected cells (Figure 2c). Infected PERK−/− cells had a comparatively mild induction of autophagy (Figure 2c), further corroborating the role of PERK in the induction of autophagy.

Furthermore, the absence of PERK also reduces the modest protection conferred by dengue against CPT toxicity in MEF cells (Figure 2d). These results were similar to those obtained for salubrinal-treated MDCK cells (Figure 1f). Hence, ER stress response (through PERK), essential for dengue-induced protection of cells, is also responsible for induction of autophagy in infected epithelial (MDCK) and fibroblast (MEF) cells. Taken together, these findings link autophagy induced by dengue infection to the PERK pathway, and its targets ATF4 and CHOP, as has also been reported in earlier studies with different models.^40, 41, 42, 43, 44^

### ATM signaling is active in infected cells and affects ER stress response, dengue-induced protection and autophagy

**Figure 3:**
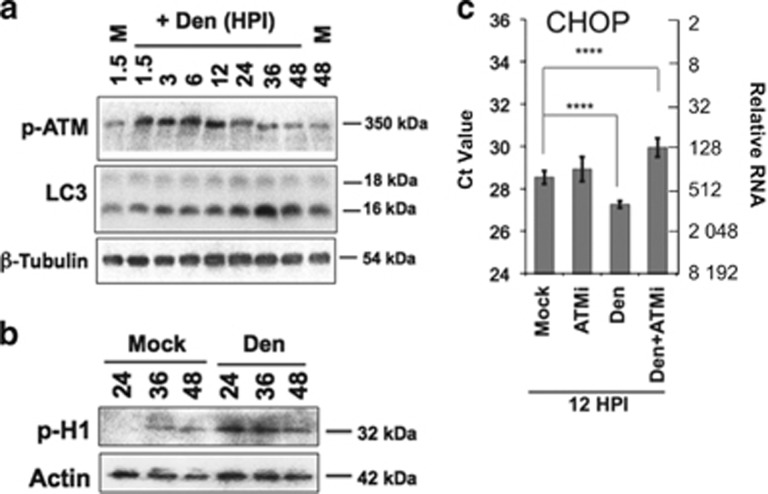
ATM kinase activation during early infection is necessary for autophagy and ER stress. (**a**) Western blot of whole-cell lysates, obtained from different times of infection, shows an increase in ATM phosphorylation (S1981) during the early stages (2–12 HPI) of infection. We also see gradual increase in LC3 lipidation from 12 up to 36 HPI, when induction reaches its highest level. *β*-Tubulin is used as loading control. (**b**) Phosphorylation of histone H1, a downstream target of ATM substrate CDK5, increases during dengue (Den) infection compared with mock-infected cells (compare Den with Mock). The maximum activation of H1 at 24 HPI corroborates the activation of ATM in the early stages of infection. Actin is used as a loading control. (**c**) CHOP RNA increases (decrease in Ct values; each unit represents a twofold difference in total RNA) during early infection (12 HPI Den). ATMi pretreatment prevents CHOP transcription at 12 HPI. Thus, an early ATM activation is responsible for ER stress induction at early stages of infection

**Figure 4:**
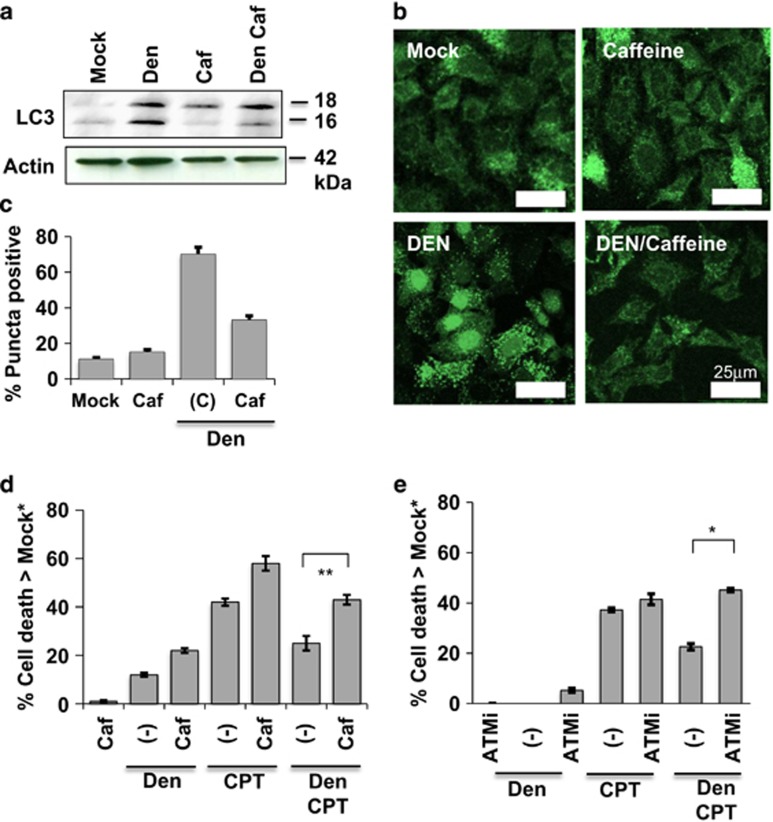
The activity of ATM is required for virus-induced autophagy and protection against CPT. (**a**) Caffeine (Caf), an inhibitor of ATM, decreases autophagy, as indicated by the decrease in the smaller LC3 band, in Dengue (Den)-infected cells (compare Den Caf with Den) after 24 h. (**b**) Caffeine (20 *μ*M) also brings down the numbers of GFP puncta in infected HeLa-GFP-LC3 after 24 h. (**c**) Quantification of GFP-LC3 punctation from the micrographs (mean of 315 cells counted per sample). (**d**) Caffeine also reduces dengue-induced protection from CPT toxicity in MDCK cells 24 h after infection. (**e**) Another ATM inhibitor (KU55933 or ATMi) also reduces dengue-induced cell protection from CPT toxicity in MDCK cells by 24 h

As ER stress response occurs downstream of ATM,^45^ we explored links between ATM activation and the ER stress induced by dengue. Although some suggest that ATM inhibits ER stress response induced by tunicamycin or ROS,^46, 47, 48^ our data indicate that in the case of dengue infection both ATM and PERK operate upstream to induction of autophagy (Figures 2a, 3 and 4). We observe an early activation of ATM (phosphorylation of serine 1981 (S1981), p-ATM) (1.5 HPI) that is sustained until 12 HPI (Figure 3a) and precedes the gradual increase of autophagy starting at 24 HPI (Figure 3a). We further evaluated ATM activity by measuring the phosphorylation of CDK5 target histone 1 that can report ATM activation.^49^ There is a significant increase in the phosphorylation of Histone 1 at 24–36 h of infection that wanes by 48 h (Figure 3b). The activation and activity of ATM before induction of autophagy indicates that ATM is upstream of infection-provoked autophagy signaling.

To determine whether the early activation of ATM by dengue is relevant to ER stress signaling, a regulator of autophagy, we assessed ER stress by transcription of CHOP. CHOP mRNA, measured by qPCR, is increased in situations of ER stress.^23^ CHOP transcription is higher in infected cells compared with mock-infected samples (Figure 3c). Inhibition of ATM by KU55933 (ATMi) suppresses increased CHOP transcription in dengue-infected cells (Figure 3c). Thus, ATM activation is necessary for the ER stress response that is activated by dengue virus. In addition, our findings with regard to CHOP support the involvement of the PERK pathway in dengue-induced autophagy.

We next examined how dengue-induced ATM activity affected autophagy after 24 h of infection. We looked at LC3 lipidation and protection against cell death (an autophagy-dependent process) in infected cells without or with 20 *μ*M caffeine, a classical inhibitor of ATM kinase. Caffeine significantly lowers LC3 lipidation in MDCK cells (Figure 4a) and autophagosome formation (Figures 4b and c) in HeLa: GFP:LC3 cells. Caffeine also increased sensitivity of infected MDCK cells to the apoptosis-inducer CPT, thereby suggesting a role for ATM signaling in cell protection (Figure 4d). We also used 5 *μ*M ATMi to measure whether ATM signaling was important to dengue-induced protection of cells; results were similar to caffeine treatment in that blockage of ATM by ATMi eliminated the protection (Figure 4e).

The increase of ATM activity during the early stages of infection suggests that ATM is upstream of virus-triggered autophagy. The fact that inhibition of ATM causes loss of dengue-induced autophagy and protection validates the role of ATM as well as that of ER stress signaling as components of autophagy signaling activated by dengue.

### At later stages of infection, PERK-dependent ROS accumulation is important for induction of autophagy

**Figure 5:**
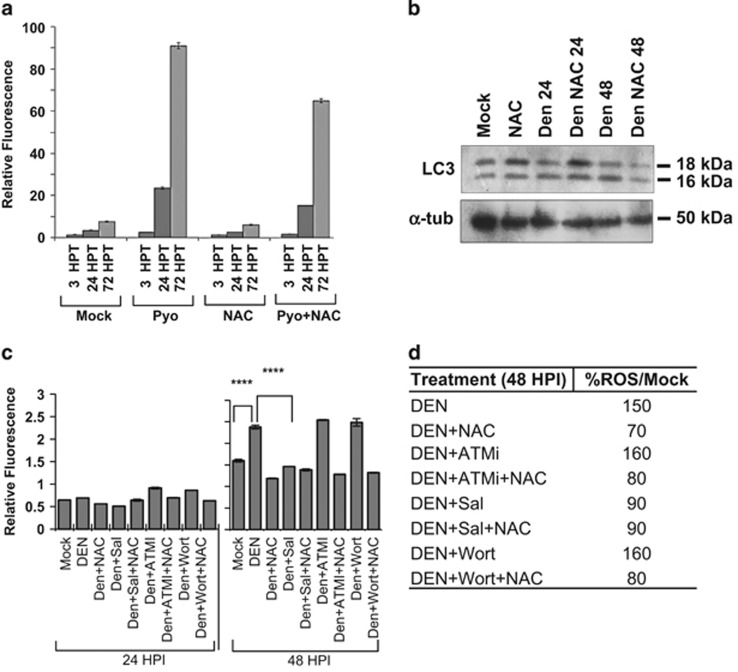
PERK-dependent ROS production has a positive effect on autophagy induction at later stages of dengue (Den) infection. (**a**) In MDCK cells, the inducer of ROS pyocyanin (100 *μ*M) gradually increases ROS (h post treatment (HPT)). ROS scavenger NAC (5 mM) partially blocks the increase in ROS. (**b**) NAC decreases dengue-induced autophagy (LC3-II, 16 kDa) at later stage (48 HPI). (**c**) Although there is little change in ROS during the first 24 HPI, by 48 HPI dengue (Den) induces an ∼30% increase in ROS (48 HPI compare Den with Mock). NAC prevents this increase, as does salubrinal (Sal) or the combination of the two. The commonly used inhibitor of autophagy wortmannin (Wort; 100 nM) and the ATM inhibitor KU55933 (ATMi) had no effect, and NAC was equally effective in their presence. ****P<0.001 for the bracketed comparisons. (**d**) The percent changes in ROS levels, compared with mock, in different samples (infected/treated) are presented in this table. NAC (a reducer of ROS) and Sal (an inhibitor of the PERK pathway) suppress the increase in ROS induced by dengue but neither ATMi nor Wortmannin (ATM and phosphatidylinositol-4, 5-bisphosphate 3-kinase (PI3K) pathway inhibitors respectively) have much impact either with or without NAC. We conclude that induction of ROS by the virus does not follow the ATM or the PI3K pathways

Activation of autophagy can be associated with the production of ROS and increased ER stress;^19^ and oxidative stress has been observed in dengue-infected cells.^50^ To determine whether dengue-induced autophagy is regulated by ROS, we first tested whether ROS are produced in MDCK cells and whether the commonly used ROS inhibitor *N-*acetylcysteine (NAC) inhibits ROS production in our system. The ROS inducer pyocyanin (Pyo) greatly increases production of ROS in MDCKs (Figure 5a), whereas the inhibitor NAC decreases Pyo-invoked ROS production even after 72 h of treatment (Figure 5a). NAC does not affect the background level of ROS (Figure 5a). Using NAC, we then asked whether ROS play a role in dengue-induced autophagy. The effect of ROS on autophagy is apparent by 48 h as shown by the decrease in LC3-II in NAC-treated infected cells (Figure 5b). The observation above is consistent with the observation that ROS increased dramatically in dengue-infected cells between 24 and 48 HPI (Figure 5c).

**Figure 6:**
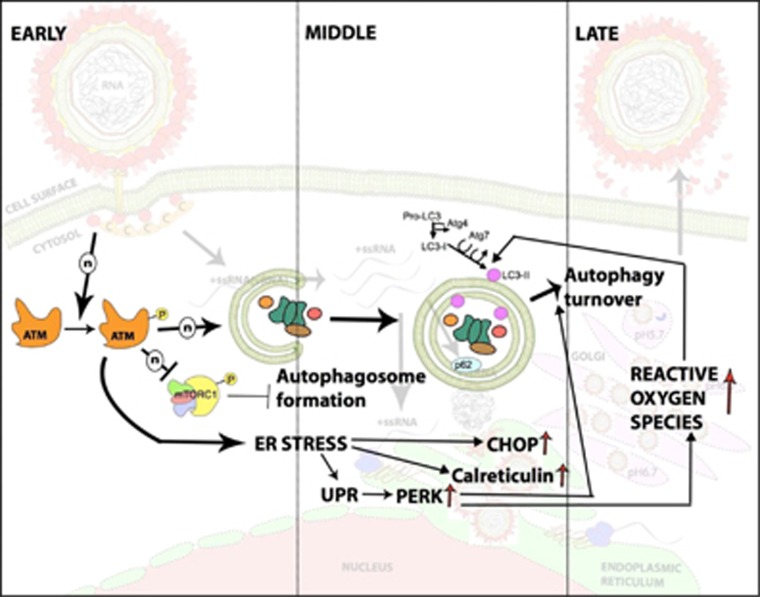
Proposed sequence of the downstream pathways activated during dengue infection. Our data suggest this sequence of stress (ATM, ER and ROS) and homeostatic (macro) autophagy pathways are activated during various stages of dengue infection. In this diagram, we highlight the different marker proteins (and associated events) we have examined in this study. The circled ***n*** signifies that the number of steps and components involved in this step of our model is still unknown. Virus infection activates autophagy by activating ATM that releases the mTORC1-derived inhibition of autophagosome formation and triggers the PERK-based ER stress pathway, furthering turnover of autophagosomes. Increase in ROS occurs late and does not participate in the protection of the cells

To examine whether ER stress, observed in early infection (Figure 1) and located upstream of autophagy (Figure 2), also affects increased ROS production late in the infection cycle (Figure 5c), we measured ROS in infected cells exposed to the ER stress inhibitor salubrinal. Salubrinal inhibits dengue-induced ROS production to similar levels as the common ROS inhibitor NAC (Figures 5c and d). NAC and salubrinal do not synergize and do not further reduce ROS (Figures 5c and d). Thus, infection-induced ROS accumulation relies on PERK-dependent signaling. These results coupled with previous observations (Figure 2) support a model whereby dengue infection triggers a later-stage accumulation of ROS, possibly through a PERK-dependent pathway, to sustain a nonlethal autophagy (Figure 6).

As ATM activity is upregulated in infected cells and affects both ER stress signaling and autophagy, we evaluated the effect of ATMi on accumulation of ROS in infected cells. ROS can activate ATM kinase.^51, 52^ However, in our system ATMi does not decrease dengue-induced ROS production (Figures 5c and d). Moreover, the commonly used autophagy inhibitor wortmannin,^53^ previously shown to inhibit dengue-induced autophagy,^5^ does not inhibit ROS production in infected cells (Figures 5c and d). However, NAC consistently decreases ROS in infected cells when either ATMi or wortmannin is present (Figures 5c and d). The inhibition of ROS by salubrinal demonstrates that the PERK pathway is important in the production of ROS during late infection.

## Discussion

### Infection activates ATM kinase that induces autophagy, leading to protection from toxins

How dengue virus regulates autophagy is poorly understood. Dengue virus 2 increases autophagosome formation and turnover. ATM kinase, known to induce autophagy in response to stress, is an upstream regulator of the mTORC1 (mammalian target of rapamycin complex 1) complex. Infection activates ATM at very early stages, without triggering cell death, followed by activation of the lysosomal system, as manifested in the high LC3 lipidation (LC3II) at a later phase of infection. ATM activation is validated by histone 1 phosphorylation. ATM inhibitor KU55933 (ATMi) transiently limits this activation, correlating with the reported half-life of ATMi.^54^ Thus, autophagy derives from ATM activation, most probably by the subsequent repression of mTORC1 complex (Figure 6), but alternative pathways may be involved as well. We examined several of these pathways in detail.

### Induction of the ER stress, especially the PERK pathway, is central to a high autophagy turnover in infected mammalian cells

Infection and viral reproduction partially depend on the metabolic and synthetic processes in the infected cells,^53, 55, 56^ including translation of dengue protein in the ER and dengue-induced alterations of the ER-Golgi network.^57, 58^ However, infected kidney cells survive and shed functional virus.^4^ ER function is closely reflected by autophagy^40^ because autophagy clears misfolded proteins and even damaged organelles.^40, 59^ Dengue infection stresses the ER through multiple pathways.^24^ Some, like the PERK pathway, are activated earlier during ER stress than others;^23^ ATF4 and CHOP transcription, in our system, increases at 12 HPI and declines thereafter, whereas transcription of GADD34 and overexpression of a general marker calreticulin persist. Calreticulin, which when overexpressed reduces the toxicity of ER stress-inducing agents,^60, 61, 62^ returns to basal levels when infected cells are treated with an inhibitor of PERK pathway, salubrinal. These kinetics differ from those reported by Pena and Harris,^24^ probably because we used different types of cells. Reducing ER stress signals by knocking out PERK produces a more modest effect than salubrinal that also affects ATF6-mediated signaling.^63^ Salubrinal inhibits increase of calreticulin, suggesting the activation of multiple ER stress pathways. Nevertheless, dengue consistently induces ER stress, and salubrinal prevents autophagy turnover as measured by p62, a protein that is sequestered and destroyed as autophagosomes fuse to lysosomes.^39^ To our knowledge, no other group has linked activation of PERK pathway and autophagy turnover during dengue infection. The inhibition of autophagy by salubrinal in PERK+/+ cells reduces intracellular dengue protein but does not prevent expression of viral protein (data not shown), indicating that the inhibitors do not prevent virus entry. Similar observations are recorded in infected PERK−/− cells that undergo lower LC3 lipidation and P62 degradation than the wild-type cells. However, inhibition of autophagy only modestly reduces production of infectious virus, again indicating that autophagy is important in enhancing, not determining, the production of infectious virus.

### Increase in ROS during infection depends upon induction of the PERK pathway

ATM is often activated in response to non-DDR events like ROS induction and inducers of autophagy.^12, 18^ As infection does not cause DDR – cell cycle arrest (data not shown) – we looked for other sources of ATM activation such as ROS production. However, in infected MDCK cells ROS induction occurs much later than ATM activation. Abolition of the ROS production (by NAC) also inhibited LC3 lipidation, suggesting that ROS induced formation of autophagosomes. The similar effects of salubrinal and NAC on ROS levels suggest a PERK-dependent ROS production during dengue infection, much like another *Flaviviridae* (hepatitis C virus (HCV)); however, this does not necessarily kill infected cells.^64, 65^ Neither KU55933 (inhibitor of ATM) nor wortmannin (inhibitor of autophagy) had any effect on the ROS levels. Therefore, ER stress signaling, but not ATM activity, regulates the increase in ROS during infection. However, the reduction of ROS does not decrease viral protein (data not shown) unlike inhibition of ATM or ER stress signaling, suggesting that autophagy at later times may not enhance virus reproduction.

### Activation of ATM kinase, and subsequent PERK activation, integrates with autophagy to protect cells from toxic assaults

The inhibition of ATM and subsequent autophagy by caffeine and KU55933 results in the loss of dengue-induced protection against other stressors. The same can be said about ER stress, as pharmacological inhibition and PERK knockout both decreased cell protection. However, ROS have no role in cell protection.

We attempted to identify key regulators of autophagy that can serve as molecular targets for drugs against dengue infection. Among the multiple autophagy-regulating and signaling pathways that are activated during dengue infection, inhibition of ER stress signaling through PERK can most effectively limit virus replication. Inhibition of ER stress is at least as effective as inhibition of ATM. Dengue-induced ER stress is a better target for inhibiting virus-induced protection and autophagy than ATM as blocking ER stress can also reduce ROS production but the net physiological result is unclear.

To our knowledge, this is the first report linking cellular stress pathway, ER stress, autophagy turnover and ROS production during dengue infection. We thus propose a sequence of events (Figure 6) suggested by our timed experiments. According to our study, ATM kinase is activated relatively early during infection. This early event leads to the repression of mTOR at the mid-stage of infection; we also have evidence of a concurrent activation of the PERK pathway by ATM kinase. Thus, ATM kinase has the ability to act as a double-edged sword, activating both ER stress and autophagy. The activated PERK pathway then rises to the occasion and upregulates ROS production and increases autophagy turnover at a later stage of infection. Although ROS modestly contribute to autophagosome formation, PERK seems to be the key player in maintaining high autophagy and production of mature and infective viral particles.

## Materials and Methods

### Cell culture and treatment

MDCK (a gift of Dr. Anastasia Gregoriades, Queens College, Flushing, NY, USA), HeLa::GFP-LC3 (provided by Guido Kroemer, Institut Gustave-Roussy, Villejuif, France), BHK (CCL-10, American Type Culture Collection (ATCC, Manassas, VA, USA)) and PERK (wild type and knockout) MEF cells (a gift of Dr. Patrizia Agostinis, Catholic University of Leuven, Belgium) were maintained in Dulbecco's modified Eagle's medium (DMEM) with 10% fetal bovine serum (FBS) and 1% penicillin/streptomycin at 37 °C under a 5% CO_2_ atmosphere. C6/36 mosquito cells (kindly provided by Adolfo Garcia-Sastre, Mount Sinai Medical School, New York, NY, USA) were maintained in Eagle's minimal essential media (10% FBS, 1 mM sodium pyruvate (S8636, Sigma, St. Louis, MO, USA), 1% nonessential amino acids (M7145, Sigma), 2 mM L-glutamine (25030-081, Invitrogen, Life Technologies Corporation, Grand Island, NY, USA), 25 units/ml fungizone (15290-018, Invitrogen) and 50 units/ml penicillin/streptomycin) at 28 °C, 5% CO_2_.

Before all infections, cells were seeded and allowed to attach overnight in maintenance media. Cells were washed with 1 × phosphate-buffered saline (PBS) before infecting at an MOI of 5 unless otherwise stated. Mock infection is treatment with virus media lacking virus and then addition with new media. Dengue-2 virus stocks were diluted with ice-cold flavivirus diluting media (1 × PBS containing 0.75% bovine serum albumin fraction V, pH 8.0). Following application of dilutions of virus, cells were incubated for 1.5 h at 37 °C (28 °C for C6/36), 5% CO_2_. Cells were then washed once with 1 × PBS, covered with maintenance media and incubated at 37 °C (28 °C for C636), 5% CO_2_ until data collection.

For expansion of stocks of dengue-2 virus (generously provided by Dr. Garcia-Sastre), subconfluent C6/36 mosquito cells were infected with virus and incubated at 28 °C for 6 days. The culture media of the infected C6/36 plates were agitated and collected, then mixed 2 : 1 with flavivirus freeze media (0.75% bovine serum albumin fraction V in 0.12 M NaCl, 0.05 M H_3_BO_3_, pH 9.0), and stored at −80 °C. Viral load of dengue stock solutions or experiments was then determined by plaque assay as described by Davis and Hardy.^66^ Briefly, confluent baby hamster kidney cells in 12-well plates were infected with serial dilutions of supernatant from virus stocks or treatments for 2 h at 37 °C before overlay with plaquing media (45% Eagle's minimal essential media; 5% FBS; 50%, 2% low melting point agarose) and incubation at 37 °C for 4 days. Agar was then removed, and cells were stained with crystal violet solution. Plaques were counted and virus titer was determined. Each sample was run in triplicate and error bars indicate 1 S.D.

When appropriate, ATM-specific inhibitor KU55933 (ATMi) (#118500, Calbiochem, San Diego, CA, USA) was applied at 5 *μ*M final concentration^67^ and ATM/ATR inhibitor caffeine (56396, Sigma) at 20 *μ*M; neither concentration leads to autophagy.^68^ Salubrinal (sc-202332, Santa Cruz Biotechnology, Dallas, TX, USA) at 3 *μ*M,^33^ and CPT (C9911, Sigma) were applied at 5–75 *μ*M final concentration,^4^ Pyo (ENZ-51010, ENZO, Farmingdale, NY, USA) at 100 *μ*M,^69^ NAC at 5 mM (ENZ-51010, ENZO),^70^ and wortmannin at 100 nM (681675, Calbiochem).^71^ In these cases, cells were incubated with inhibitors for 1 h before infection.

### Assessment of cell viability

Cells were infected and exposed to toxin (i.e., CPT) at 24 HPI, incubated for an additional 24 h at 37 °C, 5% CO_2_, collected by trypsin digestion and stained with 0.4% Trypan blue in 1 × PBS. We have previously shown a direct correlation between Trypan blue exclusion and other viability assays.^72^ Live (white) and dead (blue) cells were counted on a hemocytometer with cell viability expressed as percent dead cells greater than mock infection. In all cases, cells were incubated with toxin for 24 h before collection.

### Western blot, immunocytochemistry and cytochemistry

Cells were infected and treated as described above. At 48 HPI, cells were scraped and washed with ice-cold 1 × PBS before whole lysate proteins were collected in radio immunoprecipitation assay (RIPA) buffer and quantified using the Bio-Rad (Hercules, CA, USA) protein assay and an Ultrospec III spectrophotometer (GE Healthcare, Port Washington, NY, USA). Western blot analysis was performed by sodium dodecyl sulfate—polyacrylamide gel electrophoresis (SDS-PAGE) as described by Lin *et al.*^72^ using primary antibodies for LC3B^73^ (L7543, Sigma-Aldrich, St. Louis, MO, USA), calreticulin, phospho-S1981–ATM (all from Cell Signaling, Danvers, MA, USA) and phospho-H1 (06-597, Upstate Biotechnology, Lake Placid, NY, USA). Anti-*α-*tubulin (t6074, Sigma-Aldrich), anti-*β-*tubulin (T 5293, Sigma-Aldrich; sc9104, Santa Cruz Biotechnology) or anti-actin (A3853, Sigma) were used as loading controls. Positive signals were detected using enhanced chemiluminescence substrate (ECL; RPN2132, GE Healthcare; or 6883, Cell Signaling) and visualized using hyperfilm ECL photoradiographic film (28906835, GE Healthcare).

For immunocytochemical and cytochemical analysis, cells were seeded onto flame-sterilized glass coverslips, allowed to attach overnight and infected and treated as above. At 48 HPI, cells were washed with 1 × PBS and fixed with fresh, ice-cold 3% paraformaldehyde (04042, Fisher, Thermo Fisher Scientific, Waltham, MA, USA) for 10 min, washed once, permeabilized with 0.1 M Triton X-100, washed once and stored in the dark overnight at 4 °C in 1 × PBS with serum. Cells were then stained with 1 : 50 mouse anti-Flavi E antibody (Di-4G2-15, ATCC), 1 : 500 mouse anti-CHOP antibody, 1 : 500 rabbit anti-ATM or anti-p62 (all from Cell Signaling) followed by 1 : 500 anti-mouse IgG-AlexaFluor 488 (A11008, Invitrogen) or anti-rabbit IgG-AlexaFluor 555 (A21430, Molecular Probes, Thermo Fisher Scientific) as secondary antibody. To further ascertain the induction of autophagy by dengue virus or genes, we performed cytochemistry by measuring LC3 localization during infection as described by Kabeya *et al.*^72^ Briefly, HeLa GFP: LC3 cells were plated onto heat-sterilized glass coverslips in 35 mm plates, and then infected as described above. At 24 HPI, cells were fixed with ice-cold 3% paraformaldehyde for 10 min and rinsed once with 1 × PBS. For immunocytochemistry, cells were then embedded by Fluoromount (F4680, Sigma) and observed by confocal (Leica, Wetzlar, Germany) or fluorescence microscopy (Leitz, subsidiary of Leitz, Buffalo Grove, IL, USA). Generation of a punctate green fluorescent protein (GFP) expression pattern is indicative of LC3 translocation and autophagosome formation. Mock-infected cells were also analyzed to ensure that LC3-GFP expression alone did not cause autophagy. ImageJ software (NIH, Bethesda, MD, USA; http://imagej.nih.gov/ij/index.html) was used to count the GFP-LC3 or p62 puncta in samples.^51^ The puncta were measured using the THRESHOLD feature of ImageJ where all the background signals were successfully eliminated; the following parameters were set for counting the actual puncta (circularity: 0–1, size: 0.1–1). The total puncta in all countable cells were divided by the total number of cells (mean 315 cells, range 182–420) in each sample.

For detection of ROS in MDCK cells, the ENZO Total ROS/Superoxide detection kit (ENZ-51010, ENZO) was used according to the manufacturer's instructions. Briefly, 1 × 10^4^ cells were seeded per well in a 96-well, black wall with clear bottom plate (00913021, Corning, NY, USA) and allowed to grow overnight. The Oxidative Stress Detection (green) reagent was used to measure ROS. MDCK cells were treated with a volume of 100 *μ*l for 24 h or at a specified time with the ROS detection mix along with other treatments/compounds. The ROS/Superoxide Detection Mix was prepared by adding 4 *μ*l of the oxidative stress detection reagent in 10 ml of cultured medium. Plates were read (bottom reading) after every treatment without removing the detection/treatment mix, using a BioTek Synergy HTT Microplate reader (Biotek, Winooski, VT, USA). Standard fluorescein (excitation=490 nm, emission=528 nm) filter sets were used.

### Assessment of ER stress by qPCR

RNA from dengue-2-infected cells was collected at various times. Complementary DNA (cDNA) was then generated from extracted RNA using a Superscript III first-strand synthesis kit (18080-400, Invitrogen), following the manufacturer's protocol. Then, 1 *μ*g of cDNA was amplified by quantitative real-time PCR (qRT-PCR) in 20 ml reactions using a LightCycler FastStart DNA Master SYBR Green 1 kit (03515869001 Roche Diagnostics, Inc., Indianapolis, IN, USA), using primers specific for ATF4, CHOP (forward (Fwd): 5′-CAGAACCAGCAGAGGTCACA-3′ and reverse (Rev): 5′-CCAATTGTTCATGCTTGGTG-3′) and GADD34 (Fwd: 5′-CCAGAAACCCCTACTCATGAT-3′ and Rev: 5′-CCAATTGTTCATGCTTGGTG-3′), using a LightCycler 2.0 real time PCR machine (Roche Applied Science). We used NS4 A primers (Fwd: 5′-CGCACTGGACAACTTAGCAG-3′ and Rev: 5′-CGTGACTGTAGCCAGAAGTGTC-3′) to evaluate virus production in MDCK cells. Fold change was calculated by the following equation: 2^([dCt]), when dCt<0, or −1(2) ^ ([dCt]), when dCt>0, where dCt= difference between Ct values.

## Abbreviations

A549: human alveolar adenocarcinoma cell line
AMBRA1: activating molecule in Beclin 1-regulated autophagy
AMPK: adenosine monophosphate-activated protein kinase
ATCC: American Type Culture Collection
ATF4: cyclic AMP-dependent transcription factor 4
ATG: autophagy-related gene
ATM: ataxia telangiectasia mutated
ATMi: specific ATM inhibitor
ATR: ataxia telangiectasia and Rad3-related protein
BHK: baby hamster kidney cell line
C6/36: *Aedes albopictus* clone cell line
CDK5: cyclin-dependent kinase 5
cDNA: complementary DNA
CHOP: CCAAT/enhancer binding protein homologous protein
CPT: camptothecin
Ct: threshold cycle
DDR: DNA damage response
DMEM: Dulbecco's modified Eagle's medium
E: envelope protein
ECL: enhanced chemiluminescence substrate
eIF2*α*: eukaryotic initiation factor 2*α*
ER: endoplasmic reticulum
FBS: fetal bovine serum
Fwd: forward primer
GADD34: growth arrest and DNA damage-inducible protein 34
GFP: green fluorescent protein
HCV: hepatitis C virus
HPI: hours post infection
HT29: human colon adenocarcinoma cell line
IRE1: inositol-requiring enzyme 1
KO: knockout
KU55933: specific ATM inhibitor
LC3: microtubule-associated protein 1 light chain 3
LKB1: liver kinase B1/serine/threonine kinase 11/renal carcinoma antigen NY-REN-19
MCP: monocyte chemotactic protein
MDCK: Madin Darby canine kidney cell line
MEF: mouse embryonic fibroblast
MOI: multiplicity of infection
mTOR: mechanistic target of rapamycin/ mammalian target of rapamycin
mTORC1: mammalian target of rapamycin complex 1
NAC: *N*-acetylcysteine
NIAID: National Institute of Allergy and Infectious Diseases
NIH: National Institute of Health
NO: nitric oxide
NS4A: nonstructural protein 4A
p62: sequestosome-1/ubiquitin-binding protein
p-ATM: *phospho*-ataxia telangiectasia mutated
PBS: phosphate-buffered saline
PERK: protein kinase R-like endoplasmic reticulum kinase
Pyo: pyocyanin
qRT-PCR: quantitative real-time PCR
Rev: reverse primer
RIPA: radio immunoprecipitation assay buffer
RNS: reactive nitrogen species
ROS: reactive oxygen species
S1981: serine 1981
SDS-PAGE: sodium dodecyl sulfate–polyacrylamide gel electrophoresis
SW480: human colorectal adenocarcinoma cell line
TSC2: tuberous sclerosis complex 2/tuberin
ULK1: Unc-51 like autophagy activating kinase 1
UPR: unfolded protein response
VPS34: vacuolar protein-sorting protein 34
WIPI: WD repeat domain phosphoinositide-interacting protein
